# Altered ribosomal function and protein synthesis caused by tau

**DOI:** 10.1186/s40478-021-01208-4

**Published:** 2021-06-19

**Authors:** Harrison Tudor Evans, Deonne Taylor, Andrew Kneynsberg, Liviu-Gabriel Bodea, Jürgen Götz

**Affiliations:** Clem Jones Centre for Ageing Dementia Research, Queensland Brain Institute, The University of Queensland, Brisbane, QLD 4072 Australia

**Keywords:** FTD, Neurodegeneration, Polysome profiling, Protein synthesis, Ribosomes, Tau, Tauopathy, Translation

## Abstract

The synthesis of new proteins is a fundamental aspect of cellular life and is required for many neurological processes, including the formation, updating and extinction of long-term memories. Protein synthesis is impaired in neurodegenerative diseases including tauopathies, in which pathology is caused by aberrant changes to the microtubule-associated protein tau. We recently showed that both global de novo protein synthesis and the synthesis of select ribosomal proteins (RPs) are decreased in mouse models of frontotemporal dementia (FTD) which express mutant forms of tau. However, a comprehensive analysis of the effect of FTD-mutant tau on ribosomes is lacking. Here we used polysome profiling, de novo protein labelling and mass spectrometry-based proteomics to examine how ribosomes are altered in models of FTD. We identified 10 RPs which were decreased in abundance in primary neurons taken from the K3 mouse model of FTD. We further demonstrate that expression of human tau (hTau) decreases both protein synthesis and biogenesis of the 60S ribosomal subunit, with these effects being exacerbated in the presence of FTD-associated tau mutations. Lastly, we demonstrate that expression of the amino-terminal projection domain of hTau is sufficient to reduce protein synthesis and ribosomal biogenesis. Together, these data reinforce a role for tau in impairing ribosomal function.

**Supplementary Information:**

The online version contains supplementary material available at 10.1186/s40478-021-01208-4.

## Introduction

Tau is a neuronally-enriched microtubule-associated protein which was shown to play a role in regulating crucial molecular processes, such as synaptic plasticity, cell signalling, axonal transport, and molecular trafficking [1–4]. The pre-mRNA which encodes tau is alternatively spliced, resulting in a protein that either has zero, one, or two amino-terminal inserts (0 N, 1 N, 2 N) as well as either three or four microtubule-binding repeats (3R or 4R). As a result of this splicing, within the human brain tau exists in six major isoforms (0N4R, 1N4R, 2N4R, 0N3R, 1N3R & 2N3R) [5]. The functional domains of tau can be broadly divided into the following regions: an N-terminal projection domain (containing a phosphatase activating domain, the amino-terminal inserts and a proline-rich region), a microtubule-binding repeat domain, and a carboxy-terminal region [6]. Tau can also be altered by a remarkable number of post-translational modifications, such as phosphorylation, glycation, and ubiquitination [7].

Tau is heavily implicated in a number of neurodegenerative diseases, collectively termed tauopathies, in which tau displays an altered state of post-translational modification and forms aggregated filaments and dense tangles [8]. Tauopathies can be further subdivided into primary tauopathies, in which tau is the main pathology-inducing molecule, such as frontotemporal dementia (FTD) [9], and secondary tauopathies, in which tau pathology occurs together with amyloid-β (Aβ) deposition, as exemplified by Alzheimer’s disease (AD) [10]. Although the majority of cases of these neurodegenerative diseases are sporadic [11], a large number of familial mutations of the tau-encoding gene *MAPT,* such as P301L [12] and K369I [13] have been associated with tauopathies, e.g. FTD and Pick’s disease. Transgenic rodent models expressing these FTD-mutant forms of human tau (hTau) recapitulate many aspects of tauopathies, including synaptic loss [14] and impaired memory function [15].

Tau is shown to cause neurotoxicity and dysfunction throughout the neuron, including in axonal and dendritic projections [4], by impairing several molecular processes and hence, neuronal functions [16]. One such cellular process which has recently been shown to be altered by tau is de novo protein synthesis [17–20]. The synthesis of new proteins is vital in many neuronal processes, including, but not limited to, axonal guidance and regeneration [21], synaptic plasticity [22] and synaptic pruning [23]. Protein synthesis is also required for the formation, maintenance and extinction of long-term memories [24–26]. Recent studies have found that global protein synthesis is decreased by the expression of FTD-mutant hTau, with de novo proteomic analysis identifying a series of proteins which are altered in synthesis by FTD-mutant hTau [17, 18]. Tau has also been observed to interact with a number of proteins which interact with mRNA such as the RNA-binding protein, T cell intracellular antigen 1 (TIA1) [27] and the splicing factor proline and glutamine rich (SFPQ), also known as PTB-associated splicing factor (PSF) [28]. However, the mechanism by which tau has its effect on translation remains unclear.

One potential way by which protein synthesis is altered by FTD-mutant hTau is through alterations to ribosomes. Tau has been shown to interact with these organelles in fractionation experiments performed on human brain samples, and this interaction is thought to be stronger in AD tissue compared to healthy controls [19]. Select ribosomal proteins (RPs) have also been observed to have decreased synthesis in various mouse models of FTD, with the overall abundance of some of these RPs also being decreased in human AD and FTD brains [17, 18]. Despite this, a robust characterization of the effect of FTD-mutant hTau on RP abundance and ribosomal function has been lacking.

Here, we utilized in vitro and in vivo models of tauopathies to examine the effect of FTD-mutant hTau on RP abundance and ribosomal function. By doing so, we revealed that protein synthesis and ribosomal biogenesis are impaired by hTau expression, and we identified 11 RPs which are altered in abundance by FTD-mutant hTau expression. We also showed that these effects are facilitated by the N-terminal projection domain of hTau (*Proj-dom* hTau). Together, our results highlight that the cellular translational machinery is severely impaired in tauopathy.

## Materials and methods

### Animal ethics and primary culture

K3 mice [29] and control wild-type (WT) littermates of mixed gender were used. Mice were maintained on a 12 h light/dark cycle and provided access to food and water. All experiments were approved by and carried out in accordance with the guidelines of the Animal Ethics Committee of the University of Queensland [AEC QBI/554/17].

For primary cultures, cortices were dissected from individual K3 embryos at embryonic day 17, dissociated in a mixture of dissection media with papain, and then titrated in Neurobasal medium (Gibco, 21,103,049) supplemented with 5% fetal bovine serum (FBS), 2% B27 (Gibco, 17,504,044), 2 mM Glutamax (Gibco, 35,050,079) and 50 U/ml penicillin/streptomycin. The same number of cortical neurons was then plated into poly-D-lysine (PDL)-coated wells at 500,000 cells/well in a 12-well plate. After 72 h, the medium was replaced with Neurobasal medium with 2% B27 without FBS, and half of the medium was changed twice a week until the cells were collected. Cultures were maintained throughout at 37 °C in a humidified 5% CO_2_ incubator. At days 17 in vitro (DIV17), neurons were treated with 4 mM azidohomoalanine (AHA) (Click chemistry tools, 1066) for 16 h for fluorescent non‐canonical amino acid tagging western blot (FUNCAT-WB) analysis.

### HEK293 cell transfection and non‐canonical amino acid treatment

HEK293 cells were cultured at 37 °C in a 5% CO_2_ saturated humidity incubator in Dulbecco's modified Eagle's medium (DMEM) (Thermo Fisher, 11,965‐092) supplemented with 10% FBS and 50 U/ml penicillin/streptomycin. The same number of cells was plated for each experiment for 24 h, followed by transfection with equal amounts of plasmid using lipofectamine LTX (Thermo Fisher, 15,338,100), as per the manufacturer's instructions. All plasmids used either the pEGFP-N1 (Addgene, 6085–1) or the pCMV (Addgene, 11,153) plasmid backbone. Tau was inserted into these vectors via PCR-digestion-ligation using either XhoI (NEB, R0146) and BamHI (NEB, R3136) for pEGFP-N1, or EcoRV (NEB, R3195) and NotI (NEB, R3189) for pCMV. To examine protein synthesis, all cells were treated with 4 mM AHA for a period of 16 h before being collected. For analysis after 7 days of expression, the cells were grown in neomycin as selection marker. Cells which were analyzed via polysome profiling were not treated with AHA.

### FUNCAT-western blot and biochemical analysis

Cells were extracted in equal volumes of 1X radioimmunoprecipitation assay (RIPA) buffer (Cell Signaling, 9806), with protein concentrations determined using the bicinchoninic acid (BCA) assay (Thermo Fisher, 23,225). Newly synthesized proteins were then detected by incubating 15 µg of protein from each sample with IRDye800-DIBO (LI‐COR, 929-50,000, 1:200,000) for one hour at room temperature. Samples were then denatured via boiling 1 × Laemmli buffer, separated via SDS–PAGE and transferred to a PVDF membrane using a Turbo Transfer System (Bio‐Rad). For total protein visualization, REVERT total protein stain (LI‐COR, 926‐11,010) was used. For analysis of samples from 5 month old K3 and WT mice, proteins were extracted from one hemisphere using RIPA buffer as previously described [30]. For the detection of specific proteins, membranes were first blocked with Odyssey Tris-buffered saline (TBS) blocking buffer (LI‐COR, 927‐50,000). Membranes were then separately incubated overnight at room temperature with the following primary antibodies: Tau12 (kind gift from Dr Nicholas Kanaan, Michigan State University; 1:10,000), Tau 5 (Millipore, MAB361, 1:2,000), RPL5 (Abcam, ab86863, 1:1,000), RPS14 (ProteinTech, 16,683-1-AP, 1:1,000), RPS6 (Cell signaling, 5402, 1:500), and RPL22 (NOVUS Biologicals, NBP1-06,069, 1:1,000). Primary antibodies were added into Odyssey TBS blocking buffer. Proteins were detected using either IRDye680 anti‐rabbit IgG (LI‐COR, 926–68,071, 1:15,000), IRDye680 anti‐mouse IgG (LI‐COR, 926–68,070: 15,000) or IRDye680 anti-goat IgG (LI‐COR, 926–68,024, 1:15,000), and imaged and quantified using a LI‐COR Odyssey FC scanner. Western blots were quantified using the LI‐COR Light Studio software, with the total protein stain REVERT used for normalization.

#### Quantification of ribosomal mRNAs

For the quantification of ribosomal mRNA encoding RPL5, RPS14, RPS6 and RPL22, a semi-quantitative real-time polymerase chain reaction (qRT-PCR) protocol was adapted [31]. Briefly, total mRNA from cells expressing the constructs for either 24 h or 7 days was isolated using TRIzol lysis buffer (ThermoFisher, 15,596,026) and RNeasy kits (Qiagen, 74,004) following the manufacturer’s specification. Reverse transcription of 200 µg RNA was performed using a kit containing SuperScript III reverse transcriptase (ThermoFisher, 18,080,093) in the presence of random hexamers. The resulting cDNA was diluted 1:4, and 1 µL of the dilution was used in a qRT-PCR reaction mix containing exon-exon spanning gene-specific primers (IDT) and SYBR Green (Bio-Rad, 172–5271). The qRT-PCR was performed using a CFX384 Touch detection system (Bio-Rad) and the results were evaluated using the manufacturer’s software, with amplification specificity being confirmed by analyzing the melting curve specificity. The change in mRNA of the genes of interest was assessed against Gapdh mRNA, with Gapdh proteins being confirmed as an adequate house-keeping gene in our proteomic analysis. The qRT-PCR quantification was performed using the ΔΔCt method.

### Polysome profiling

Polysome profiling was performed as previously described but with minor modifications [32]. Briefly, transfected HEK293 cells were treated with 10 mg/mL cycloheximide (CHX) (Sigma-Aldrich, 01,810) for 3 min. Taking care to avoid RNAse contamination, cells were then placed on ice and washed in PBS with 10 mg/mL CHX, after which they were lysed in fresh lysis buffer (50 mM KCl, 20 mM Tris.HCl pH 7.5, 10 mM MgCl_2_, 1% Triton X100, 1 mM 1,4-dithiothreitol (DTT), 0.5% w/v sodium deoxycholate, 1X protease and phosphatase inhibitors, 10 mg/mL CHX, 1:1000 RNAse OUT inhibitor (Invitrogen, 10,777–019)). Samples were then centrifuged for 5 min at 13,000 × g at 4 °C. The supernatant was then loaded onto a 10–50% sucrose linear gradient (50 mM KCl, 20 mM Tris-HCl pH 7.5, 10 mM MgCl_2_) created using the BioComp gradient station (BioComp, 153). The 40S and 60S ribosomal subunits, along with monosomes and polysomes were then separated via centrifugation at 235,000 × g at 4 °C for 2 h and detected using the BioComp TRIAX flow gradient collection system (BioComp, FC-1-26). Abundance of the various ribosomal complexes was quantified by calculating area under the curve (AUC) for these regions of the polysome profile.

### Nano‐liquid chromatography tandem mass spec (nano‐LC MS/MS) label free quantification

In preparation of analysis via nano-LC MS/MS, samples were reduced with 5 mM DTT at 60 °C for 30 min, followed by alkylation in 10 mM iodoacetamide (IAA) for 15 min in the dark at 25 °C, with excess IAA being quenched with an equivalent amount of DTT. Samples were then acidified with 12% orthophosphoric acid, then with S-trap loading buffer (90% methanol, 100 mM tetraethylammonium bromide (TEAB), pH 7.1). Proteins were then loaded onto an S-trap micro (Protifi), before being digested with 1.4 μg Tryspin in 100 mM TEAB for 1.5 h at 37 °C. After digestion, peptides were eluted from the trap with 100 mM TEAB, 0.2% formic acid, then 50% acetonitrile 0.2% formic acid, followed by lyophilization. Peptides were reconstituted with 30 μL of 0.1% formic acid, with 6μL of sample being loaded for injection. Peptide samples were injected (6 μl) onto the peptide trap column and washed with loading buffer for 10 min. The peptide trap was then switched in line with the analytical nano-LC column. Peptides were eluted from the trap onto the nano-LC column and separated with a linear gradient of 3.5% mobile phase B to 25% mobile phase B over 70 min at a flow rate of 600 nl/min and then held at 85% B for 8 min prior to re-equilibration.

The column eluent was directed into the ionization source of the mass spectrometer operating in positive ion mode. Peptide precursors from 350 to 1850 m/z were scanned at 60 k resolution. The 20 most intense ions in the survey scan were fragmented using a normalized collision energy of 33 with a precursor isolation width of 1.3 m*/z*. Only precursors with charge state + 2 to + 5 were subjected to MS/MS analysis. The MS method had a minimum signal requirement value of 4.3 × 10^4^ for MS2 triggering, an AGC target value of 3 × 10^6^ and a maximum ion injection time of 45 ms. MS2 scan resolution was set at 3 × 10^4^, an AGC target value of 1 × 10^5^ and a maximum injection time of 70 ms. MS/MS scan resolution was set at 3 × 10^4^ and dynamic exclusion was set to 30 s. The mass spectrometric data files were searched using Proteome Discoverer (Thermo, Version_2.1) embedded with search engine SequestHT against *Mus musculus* (17,002 sequences, accessed November 2019, https://www.uniprot.org/*)* protein sequences downloaded from the UniProt database. Label free quantification proteomic data were normalised to total protein abundance in each sample. Technical replicates were averaged for each sample. Fold-change to WT samples were then compared via Student’s t-test with proteins having a *p* value ≤ 0.05 and an absolute fold-change ≥ 1.5 being classified as being differentially expressed.

### Bioinformatic analysis

Network analysis was performed as previously described [24], with minor modifications. Briefly, data from the label-free quantitative mass spectrometry were mapped to the STRING protein query database for *Mus musculus* using Cytoscape (v3.8.2) and the edge-weighted spring-embedded layout. A STRING confidence of interaction score cut-off of 0.7 was used. Clusters of regulated proteins were identified using Molecular Complex Detection (MCODE) [33]. The proteins in these clusters where then analyzed using the REACTOME database.

### Statistical analysis

All statistical analysis was performed on samples run at least in experimental triplicate, as detailed in the Figure legends. Statistics was performed in GraphPad Prism 7.0 software, using either one‐way ANOVA or Student's t‐test, with Tukey's multiple comparison test (MCT), as appropriate. All values are given as mean ± standard error of the mean (SEM). Significance was defined as *p < 0.05, **p < 0.01, ***p < 0.001, ****p < 0.0001.

## Results

### Select ribosomal proteins are altered in abundance in K369I hTau expressing primary cortical neurons

**Fig. 1:**
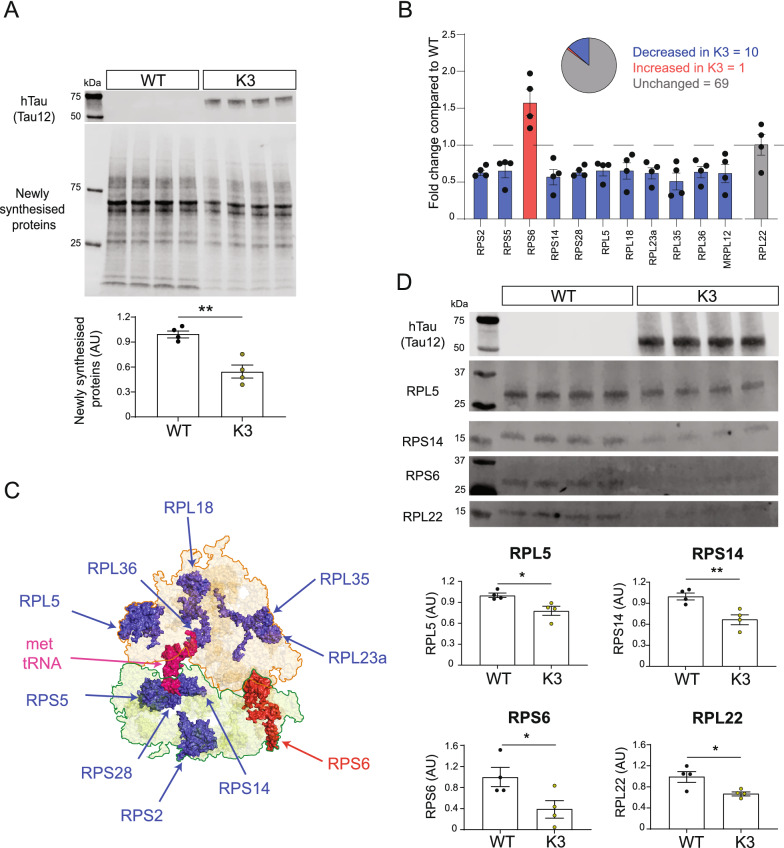
Proteomic analysis identifies altered abundance of select ribosomal proteins in K369I-hTau expressing primary neurons **a** FUNCAT-WB analysis confirms that global protein synthesis is decreased in K3 primary neurons compared to WT littermates. Primary cortical neurons were cultured from individual K3 and WT pups, before being treated with 4 mM AHA for 16 h at DIV17. The amount of new protein synthesis during this 16 h window was then quantified by using FUNCAT-WB to fluorescently tag AHA-labelled proteins. The human-tau specific Tau12 antibody was used to identify K3 positive pups. FUNCAT signal was normalised to the total protein stain REVERT. n = 4 animals, unpaired t-test. **b** 80 ribosomal proteins (RPs) were quantified in K3 and WT primary neurons using untargeted, label-free 1D-LC MS/MS analysis. Of these, 11 RPs (RPS2, RPS5, RPS14, RPS28, RPL5, RPL18, RPL23a, RPL35, RPL36 and MRPL12) were found to be significantly decreased (FC ≤ 0.66, * p*≤ 0.05) in K3 primary neurons compared to WT littermates, whereas 1 RP (RPS6) was significantly increased (FC ≥ 1.5, * p* ≤ 0.05) in K3 primary neurons. RPL22 is shown as an example of an RP unchanged in abundance between K3 and WT primary neurons. n = 4 animals, unpaired t-test. **c** Surface representation of the ribosome complex (PDB: 4ug0) with RPs found to have significantly decreased abundance in K3 primary neurons (FC ≤ 0.66, * p* ≤ 0.05) shown in blue, and RPS6, which displayed significantly increased abundance (FC ≥ 1.5, * p* ≤ 0.05) in K3 primary neurons shown in red. Unchanged RPs belonging to the 60S subunit are shown in orange, whereas unchanged RPs which form part of the 40S subunit are shown in green. **d** Western blot analysis reveals that the abundance of candidate RPs is decreased in 5 month-old K3 mice compared to WT littermates. Protein abundance was normalised to the total protein stain REVERT. n = 4 animals, unpaired t-test

To explore the effect of FTD-mutant hTau on both global translation and ribosomal subunit abundance, we utilized primary cortical neurons cultured from the K3 mouse model of FTD [29, 34]. We previously reported decreased protein synthesis in these mice that was correlated with tau pathology [17]. Here, we extend these findings and show that overall de novo protein synthesis is also decreased in K3 primary neuronal cultures (Fig. 1a). This was achieved by using non-canonical amino acid (NCAA) labelling, in which de novo synthesised proteins were labelled with the methionine surrogate azidohomoalanine (AHA). Newly synthesized proteins containing AHA were then fluorescently labelled through the reaction of the azide-group of AHA with an DIBO-conjugated fluorophore before being analyzed via western blot in a technique known as fluorescent non‐canonical amino acid tagging followed by western blot analysis (FUNCAT-WB) [35]. Cortical neurons were cultured from individual pups until DIV17, before being treated with AHA for 16 h before FUNCAT-WB analysis, with the human tau-specific Tau12 antibody being used to distinguish K3 and WT pups. The FUNCAT-WB signal was significantly reduced in K3 samples compared to WT controls (Fig. 1a), showing that protein synthesis was decreased in K3 primary neurons.

Next, we used label-free quantitative tandem mass spectrometry to determine if ribosomal protein abundance was altered in K3 primary neurons. This allowed us to quantify the levels of 80 ribosomal proteins, including 72 of the ≈80 eukaryotic cytosolic ribosomal proteins and 8 mitochondrial RPs (Fig. 1b, Additional file 4). Of these proteins, 11 were altered in abundance (|FC|≥ 1.5, * p* ≤ 0.05), with 10 showing decreased abundance (RPS2, RPS5, RPS14, RPS28, RPL5, RPL18, RPL23a, RPL35, RPL36 and MRPL12) in K3 primary neurons compared to WT littermates (Fig. 1b). RPS6 was the only ribosomal protein that was increased in K3 compared to WT samples (Fig. 1b). Interestingly, these alterations in RP abundance in K3 mice appeared to be evenly distributed across both the 40S and 60S ribosomal subunit (Fig. 1c). Additionally, we observed that the abundance of many eukaryotic initiation factors involved in translation (eIF3E, eIF4G2, eIF4B, eIF3G and eIF5) was also decreased in K3 primary neurons (Additional file 1: Fig S1, Additional file 5).

To determine if these changes in RP abundance were also found in vivo and, thus, associated with a more advanced pathology, we quantified the abundance of a subset of representative RPs in brain homogenates from K3 and WT mice at 5 months of age, when tau pathology is robust [36] (Fig. 1d). By western blotting, we found that in K3 brain, the abundance of RPS14 and RPL5 was also decreased (Fig. 1d), recapitulating our findings from primary neuronal experiments. Interestingly, unlike in primary neurons, the abundance of RPS6 was decreased in K3 mice (Fig. 1d). Furthermore, the abundance of RPL22 was decreased in K3 mice (Fig. 1d), unlike in K3 primary neurons, where its abundance was unaltered (Fig. 1b). This suggests that the extended exposure to pathogenic tau in in vivo models of tauopathy may lead to a more robust decrease in select RP levels.

### FTD-mutant hTau expression decreases protein synthesis and ribosome complex formation in transfected HEK293 cells

**Fig. 2:**
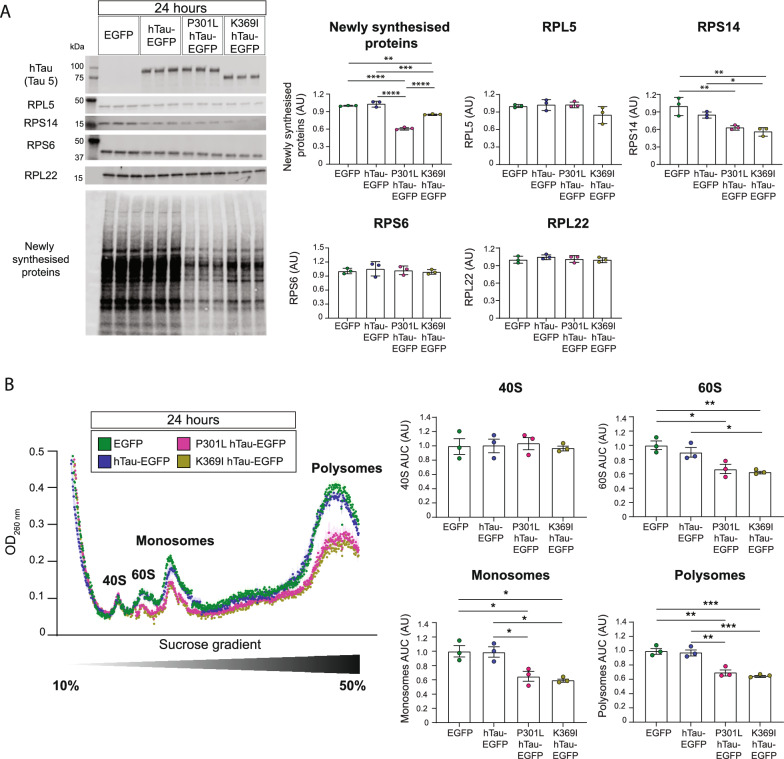
24 h of FTD-mutant-hTau expression reduces protein synthesis and impairs ribosomal biogenesis in HEK293 cells. **a** FTD-mutant tau expression reduces protein synthesis and abundance of RPS14 over a period of 24 h. HEK293 cells were transfected with either 2N4R hTau-EGFP, 2N4R P301L-hTau-EGFP, 1N4R K369I-EGFP or EGFP control and then treated with 4 mM AHA at 8 h post-transfection. At 24 h, AHA labelled de novo synthesised was quantified via FUNCAT-WB, with both P301L-hTau and K369I-hTau expressing cells showing reduced protein synthesis compared to EGFP and non-mutant hTau expressing cells. The abundance of RPS14 was changed in FTD-mutant tau expressing cells; the abundance of other RPs, such as RPL5 and RPS6, was unchanged at 24 h. Protein abundance was normalised to the total protein stain REVERT. n = 3 wells, one-way ANOVA, Tukey’s MCT. **b** Polysome profiling reveals that FTD-mutant tau expression reduces polysome, monosome, and 60S ribosomal subunit abundance after 24 h. HEK293 cells transfected with EGFP, 2N4R hTau-EGFP, 2N4R P301L-hTau-EGFP or 1N4R K369I-EGFP were treated with 100 ug/mL CHX for 5 min in order to prevent the dissociation of bound ribosomes from mRNA. Following lysis, samples were separated on a 10–50% linear sucrose gradient via ultracentrifugation. Absorbance at 260 nm was used to detect the presence of the 40S and 60S ribosomal subunits, along with monosome and polysomes, with the area under the curve (AUC) being used for quantification. Both P301L-hTau and K369I-hTau expressing cells showed reduced levels of polysomes, monosomes, and the 60S ribosomal subunit compared to EGFP and non-mutant hTau expressing cells. n = 3 wells, one-way ANOVA, Tukey’s MCT

Building upon our findings in K3 primary neurons, we next sought to determine if expression of FTD-mutant hTau, initially over a period of 24 h, was sufficient to decrease protein synthesis and alter RP abundance. We therefore utilised a HEK293 cell model in which we induced the expression of either non-mutant hTau or mutant forms of hTau associated with inherited tauopathy (K369I-hTau and P301L-hTau) [5] for 24 h, with newly synthesised proteins again being labelled with AHA (Fig. 2a). For non-mutant hTau and P301L-hTau expressing cells, the full length (2N4R) isoform of hTau was used, whereas in cells transfected with K369I-hTau, the 1N4R isoform was used as this matches what is expressed in K3 mice [29]. All forms of hTau were expressed by fusion to EGFP, with EGFP-only expressing cells serving as an expression control.

FUNCAT-WB analysis revealed that 24 h of FTD-mutant hTau expression resulted in significantly decreased overall de novo protein synthesis, with both P301L-hTau and K369I-hTau expressing cells showing a decreased FUNCAT-WB signal compared to both hTau and EGFP expressing cells (Fig. 2a). Western blots were performed to examine the abundance of a subset of RPs in these transfected cells. Interestingly, despite the large decrease in global protein synthesis, only the abundance of RPS14 was decreased in the FTD-mutant hTau expressing cells, with the abundance of both RPL5 and RPS6 being unchanged at this time-point (Fig. 2a). The abundance of RPL22 was also not altered in hTau or FTD-mutant hTau expressing cells, a finding similar to what was observed in K3 primary neurons (Fig. 2a). Semi-quantitative real-time polymerase chain reaction (qRT-PCR) analysis revealed that the abundance of mRNAs encoding RPS14, RPL5 and RPL22 was unchanged after 24 h of hTau or mutant tau expression (Additional file 2: Fig. S2A).

Given that we observed a large decrease in protein synthesis in HEK293 cells expressing K369I-hTau or P301L-hTau, we next sought to examine if FTD-mutant hTau expression altered ribosomal complex formation or ribosomal biogenesis. For this we used polysome profiling, a technique in which polysomes, monosomes, and the 60S and 40S ribosomal subunits are separated on a linear sucrose gradient and their abundance is quantified by measuring their absorbance at 260 nm, which detects the rRNA bound within these ribosomal complexes. We found that 24 h post-transfection, the abundance of both polysomes and monosomes were decreased in K369I-hTau and P301L-hTau expressing cells compared to the EGFP control (Fig. 2b), which is consistent with our previous observation that protein synthesis is decreased by FTD-mutant hTau expression. Interestingly, the abundance of the 60S ribosomal subunit was also decreased in these cells, suggesting that FTD-mutant hTau expression may impair ribosomal biogenesis (Fig. 2b), which precedes ribosome-mediated translation. We also demonstrated that K369I-hTau expression decreased polysome, monosome, and 60S subunit abundance regardless of whether the 1N4R or 2N4R isoform was used (Additional file 3: Fig S3).

### Select ribosomal protein abundance is decreased by both hTau and FTD-mutant hTau after 7 days of expression in HEK293 cells

**Fig. 3:**
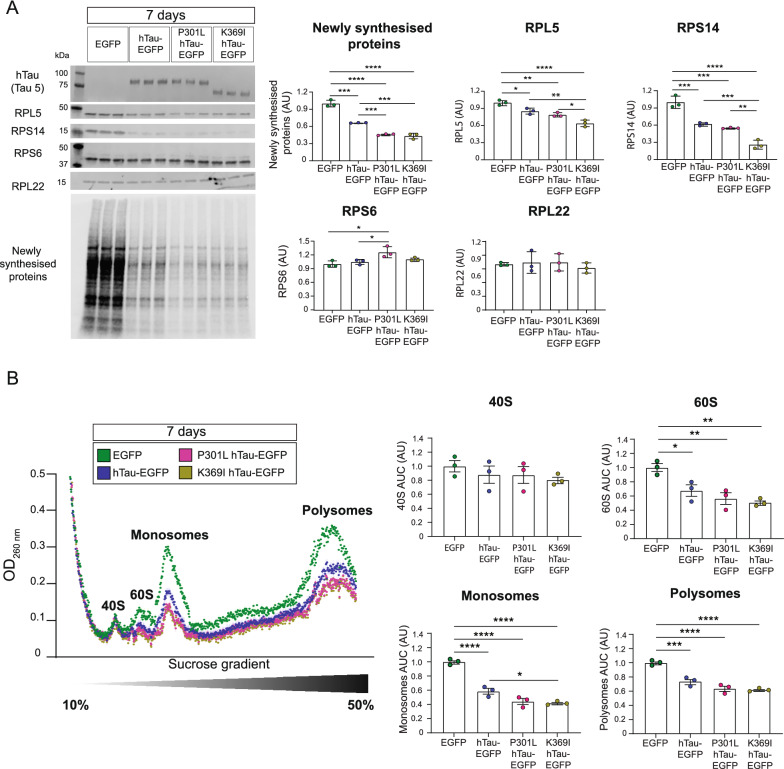
7 days of hTau and FTD-mutant hTau expression alter RP abundance, protein synthesis and ribosomal biogenesis. **a** 7 days of hTau overexpression decreases protein synthesis and alters the abundance of select ribosomal proteins (RPs) in HEK293 cells. Cells expressing EGFP, hTau-EGFP, P301L-hTau-EGFP or K369I-hTau-EGFP were selected by using neomycin. Newly synthesised proteins were labelled for 16 h with AHA, before collection at 7 days post transfection. FUNCAT-WB showed that protein synthesis was decreased by hTau expression, with this decreasing being greater in FTD-mutant hTau expressing cells. hTau, P301L-hTau, and K369I-hTau expression also lead to decreased abundance of RPL5 and RPS14. The abundance of RPS6 was increased in P301L-hTau expression cells compared to EGFP controls. Protein abundance was normalised to the total protein stain REVERT. n = 3 wells, one-way ANOVA, Tukey’s MCT. **b** Polysome, monosome and 60S ribosomal subunit abundance is decreased by 7 days of hTau expression in HEK293 cells. Transfected cells were treated with 100 ug/mL CHX for 5 min in order to prevent the dissociation of bound ribosomes from mRNA and following lysis, ribosomal complexes were separated on a 10–50% linear sucrose gradient via ultracentrifugation. 40S and 60S ribosomal subunit, along with monosome and polysomes, were detected via their absorbance at 260 nm, with the area under the curve (AUC) of these peaks being used for quantification. The abundance of the 60S ribosomal subunit, monosomes and polysomes was decreased in all three hTau expressing groups. n = 3 wells, one-way ANOVA, Tukey’s MCT

Given the relatively long half-lives of ribosomal proteins [37] and the limited changes observed in select RP abundance after 24 h of FTD-mutant hTau expression, we next sought to examine whether RP abundance was altered after 7 days of FTD-mutant hTau expression in HEK293 cells. We found that changes in RP abundance were more pronounced at this time point, with RPL5 and RPS14 levels being decreased by FTD-mutant hTau and RPS6 abundance being increased in P301L-hTau expressing cells (Fig. 3a). Interestingly, unlike at the 24 h time-point, after 7 days of expression non-mutant hTau also decreased levels of RPL5, RPS14, as well as decreasing overall de novo protein synthesis as measured by FUNCAT-WB (Fig. 3a). These decreases, however, were less pronounced than for FTD-mutant hTau expressing cells, suggesting that the FTD-associated mutations analysed in this study have an accentuated effect on the interaction between hTau and ribosomal function (Fig. 3a). No significant change was observed of RP transcript levels in FTD-mutant hTau expressing cells after 7 days (Additional file 2: Fig S2B).

With these findings in mind, we next sought to determine if non-mutant hTau expression also altered ribosomal complex formation after 7 days. Using polysome profiling, we demonstrated that the abundance of polysomes, monosomes, and the 60S ribosomal subunit was decreased in all hTau expressing groups compared to EGFP transfected controls (Fig. 3b). Interestingly, we found that FTD-mutant hTau had a greater effect on monosome abundance compared to non-mutant hTau (Fig. 3b). Together, these data suggest that, as with FTD-mutant hTau, elevated levels of hTau also alter the biogenesis of the 60S ribosomal subunit.

### Expression of the N-terminal projection domain of hTau is sufficient to decrease protein synthesis and ribosome complex formation

**Fig. 4:**
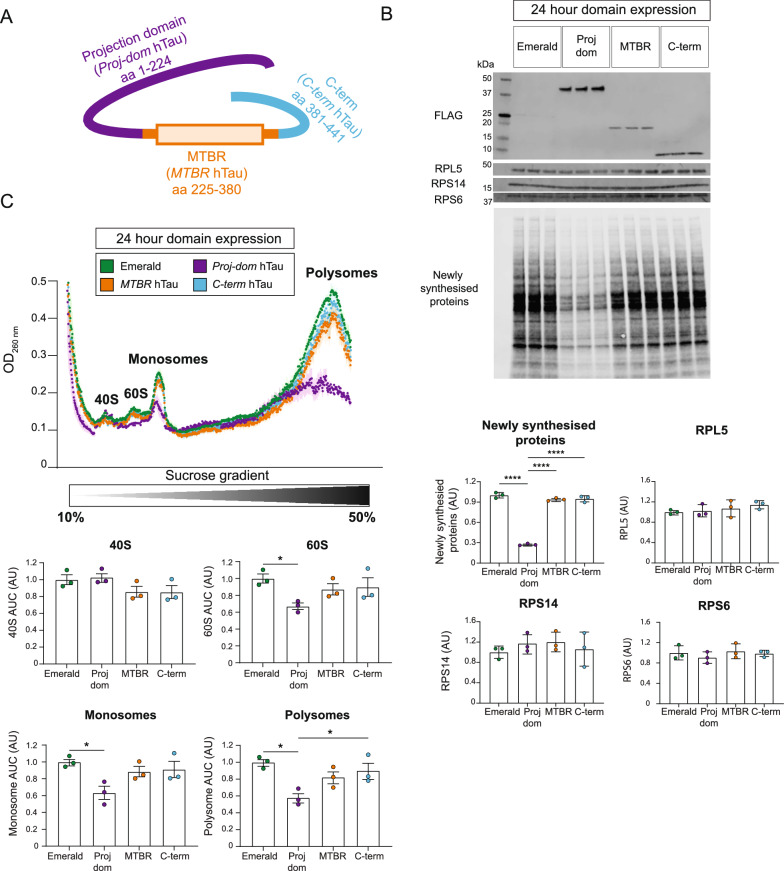
Expression of the N-terminal projection domain of hTau dramatically decreases protein synthesis and ribosomal biogenesis. **a** Schematic of the hTau regions separately expressed in HEK293 cells and investigated in the study: hTau’s N-terminal projection domain (AA 1-AA 224, *Proj-dom* hTau), microtubule binding region (AA 225-AA 380, *MTBR* hTau), and carboxy-terminal region (AA 381- AA 441, *C-term* hTau). **b** Protein synthesis is reduced by the expression of the N-terminal projection domain of hTau in HEK293 cells. 4 mM AHA was used to label newly synthesised proteins for 16 h in cells expressing either Emerald, *Proj-dom* hTau, *MTBR* hTau, or *C-term* hTau. FUNCAT-WB analysis revealed that de novo protein synthesis was only decreased in *Proj-dom* hTau expressing cells after 24 h of expression. The abundance of RPL5, RPS14, RPS6 and RPL22 was found to be unaltered in these cells. Expression of the various domains of hTau was detected using the FLAG tag fused to these domains. Protein abundance was normalised to the total protein stain REVERT. n = 3 wells, one-way ANOVA, Tukey’s MCT. **c** Polysome profiling reveals that the abundance of polysomes, monosomes and the 60S ribosomal subunit is decreased by 24 h of *Proj-dom* hTau expression. Transfected cells were treated with 100 ug/mL CHX for 5 min in order to prevent the dissociation of bound ribosomes from mRNA, and following lysis, ribosomal complexes were separated on a 10–50% linear sucrose gradient via ultracentrifugation. 40S and 60S ribosomal subunits, along with monosome and polysomes were detected via their absorbance at 260 nm, with the area under the curve (AUC) of these peaks being used for quantification. The abundance of the 60S ribosomal subunit, monosomes and polysomes was only decreased in *Proj-dom* hTau expressing cells. n = 3 wells, one-way ANOVA, Tukey’s MCT

Given our observation that hTau and FTD-mutant hTau can decrease protein synthesis and the abundance of particular ribosomal complexes, we next sought to determine which regions of hTau were involved in this. Thus, we expressed various truncated domains of hTau in HEK293 cells, including the N-terminal projection domain of hTau (*Proj-dom* hTau*,* aa1- 224, which contains tau’s phosphatase activating domain, the amino-terminal inserts and proline-rich region); the microtubule-binding domain (*MTBR* hTau, aa225-380); and the carboxy-terminal region of tau (*C-term* hTau, aa381-441) (Fig. 4a). Cells transfected with these constructs were analysed using FUNCAT-WB and polysome profiling.

Interestingly, whereas full-length non-mutant hTau required longer periods of expression to alter protein synthesis, *Proj-dom* hTau was able to considerably decrease protein synthesis after only 24 h of expression (Fig. 4b). However, unlike FTD-mutant hTau, *Proj-dom* hTau expression did not alter RP abundance at this time point (Fig. 4b). Using polysome profiling, we also observed that *Proj-dom* hTau expression resulted in a decreased abundance of polysomes, monosomes and the 60S ribosomal subunit when compared to an Emerald-expressing control (Fig. 4c). Expression of *MTBR* hTau and *C-term* hTau, on the other hand, had no observable effect on protein synthesis, RP abundance or ribosomal complex formation (Fig. 4b, c).

## Discussion

Aberrant changes in the microtubule-associated protein tau can severely impair the fundamental cellular process of protein synthesis [17–19]. These studies have also suggested that changes in the synthesis of ribosomal proteins (RPs) may be, in part, responsible for the observed changes in protein synthesis. Despite this, until present, a robust analysis of how RP abundance and ribosomal function are altered in models of tauopathy has been lacking. In the current study, we utilised proteomics, mRNA quantification, non-canonical amino acid labelling and polysome profiling to examine how the translational machinery is altered by the presence of various forms of human tau. By quantifying the levels of 72 of the ≈80 eukaryotic RPs, we revealed that 11 of these RPs were altered in their abundance in primary neurons cultured from the K3 mouse model of FTD, with 10 of these RPs showing decreased levels. By analysing a subset of these dysregulated RPs via western blotting, we revealed that RP abundance was also altered in both adult K3 mice and HEK293 cells transfected with various forms of human tau (hTau). Using polysome profiling, we also determined that FTD-mutant hTau expression can impair the biogenesis of the 60S ribosomal subunit and decrease the abundance of polysomes and monosomes. Lastly, we demonstrated that expression of the N-terminal projection domain of hTau alone was sufficient to impair protein synthesis and ribosomal biogenesis.

The eukaryotic ribosome is a large RNA–protein complex which contains two ribosomal subunits: the large 60S and the small 40S subunit. Together, these subunits consist of the 28S, 18S, 5.8S and 5S rRNAs and approximately 80 RPs [38]. While previous work has suggested that RP synthesis is decreased in models of FTD [17, 18], the effect of FTD-mutant hTau on overall RP abundance has remained unclear until now. In this study, we demonstrated that K369I-hTau expressing primary neurons had decreased abundance of 10 RPs (RPS2, RPS5, RPS14, RPS28, RPL5, RPL18, RPL23a, RPL35, RPL36 and MRPL12) (Fig. 1b). We also confirmed that the abundance of RPL5 and RPS14 was decreased in 5 month-old K3 mice (Fig. 1d).

Additionally, we observed an increased abundance of RPS6 in both K3 primary neurons and HEK293 cells expressing P301L-hTau for 7 days (Figs. 1b, 3a). Increased RPS6 signalling has previously been observed in other models of tauopathy, with the phosphorylation of RPS6 being increased when hTau was co-expressed with the tyrosine kinase, Fyn [20]. Interestingly, we observed that, unlike in vitro (Figs. 1b, 2a, 3a, 4a), in 5 month-old K3 mice compared to their WT littermates, RPS6 was decreased (Fig. 1d). This, together with a similar decrease in RPL22 in K3 mice, suggests that prolonged FTD-mutant hTau expression may lead to a greater decline in ribosomal protein abundance. Changes in RP abundance have also been suggested to occur in Parkinson’s disease [39], AD [40] and spinal muscular atrophy [41]. Taken together with our findings this would suggest that alterations in RP abundance are a hallmark of neurodegenerative diseases.

One potential mechanism for how these changes in RP abundance are caused is through alterations to the mammalian target of rapamycin (mTOR) signal transduction pathway, which can regulate the translation of select mRNAs, including many of the mRNAs which encode RPs [42–44]. Alterations in mTOR signaling have been observed in AD [45], and the synthesis of mTOR was found decreased in K3 mice [17]. These changes in mTOR may contribute to the alteration in RP abundance we observed here in in vitro models of tauopathy.

We also found that additional proteins involved in translation were decreased in abundance in K3 primary neurons. This included the eukaryotic initiation factors (eIFs) eIF3E, eIF4G2, eIF4B, eIF3G and eIF5, the eukaryotic peptide chain release factor eTF1, as well as proteins involved in pre-mRNA splicing such as ELAV-like protein 1 and splicing factor 3A (Additional file 1, Additional file 5).

Our polysome profiling analysis revealed that various forms of hTau can decrease the levels of the 60S ribosomal subunit, suggestive of an impairment in 60S ribosomal biogenesis. This decrease in 60S subunit abundance likely contributed to the decrease in monosomes and polysomes resulting from aberrant hTau expression, although tau may also decrease monosome and polysome numbers through mechanisms independent of altering ribosomal biogenesis. Interestingly, both FTD-mutant hTau and *Proj-dom* hTau decreased protein synthesis and 60S biogenesis already after 24 h of expression, without detectably altering the abundance of RPs belonging to the 60S ribosomal subunit (Figs. 2a, b, 4b, c). Furthermore, despite the decreased abundance of RPs being evenly distributed across the 60S and 40S subunits, cells which expressed either hTau or FTD-mutant hTau for a period of 7 days only showed decreased levels of the 60S ribosomal subunit, with the abundance of the 40S subunit being unaltered (Fig. 3a,b). Taken together, these findings would suggest that hTau may exert its effect on protein synthesis and 60S ribosomal subunit biogenesis independently of altering RP abundance.

We also observed that the effect of hTau upon protein synthesis, RP abundance and ribosomal complex formation was not unique to FTD-mutant hTau. After longer periods of expression non-mutant hTau was also able to decrease protein synthesis and the abundance of RPL5, RPS14, polysomes, monosomes, and the 60S subunit (Fig. 3a,b). This is aligned with previous findings showing that hTau alters polysome abundance when incubated with yeast ribosomes [46]. However, we also found that the effect of non-mutant hTau on protein synthesis, RP abundance and ribosomal complexes was less pronounced than for FTD-mutant hTau (Fig. 3a,b) and that, unlike FTD-mutant hTau, alterations to the protein translational machinery were only detectable after 7 days of non-mutant hTau overexpression. These results suggest that the effect of hTau on protein synthesis and ribosomes is accelerated by the presence of FTD-associated mutations.

Interestingly, unlike full-length non-mutant hTau, expression of the *Proj-dom* hTau for 24 h was sufficient to decrease protein synthesis and the abundance of polysomes, monosomes, and the 60S ribosomal subunit (Fig. 4b,c). This demonstrates that the ability for hTau to decrease protein synthesis and ribosomal complex formation is facilitated by its N-terminal projection domain.

One possible explanation for the observed differences in the abilities of FTD-mutant hTau, non-mutant hTau, and *Proj-dom* hTau to impact the protein translation machinery is through changes to the conformation of tau. Tau has been claimed to exist in a ‘paperclip-like’ conformation, by which the N-terminal projection domain and the carboxy-terminal region fold back toward the microtubule-binding region (Fig. 4a) [47]. When tau is bound to microtubules, the interaction between these different regions of tau is thought to be stronger, leading to a more ‘closed’ conformation of the N-terminal projection domain [48]. However, when tau is not bound to microtubules or is phosphorylated at specific sites, the N-terminal projection domain is thought to interact with other proteins more easily [48, 49].

It is therefore possible that in the case of non-mutant hTau, the majority of tau exists in this more ‘closed’ conformation at 24 h, preventing the N-terminal projection domain of tau from interacting (either directly or indirectly) with ribosomes. As a result, we only observed decreases in protein synthesis and ribosomal complex formation after 7 days of non-mutant hTau expression, when sufficient tau was in a more ‘open’ conformation, either through changes in phosphorylation or microtubules saturated with tau. However, when expressing only the N-terminal projection domain of hTau, this domain is thought to be fully exposed and therefore maybe more able to affect ribosomes. This may in turn explain the pronounced decreases in protein synthesis and ribosomal complex formation observed after only 24 h of *Proj-dom* hTau expression. In regard to FTD-mutant hTau, mutations such as P301L and K369I are thought to alter the conformation of tau [50] and decrease microtubule binding [51], which may increase the ability of tau to aberrantly interact with other molecules [48]. This change in conformation may explain why the N-terminal projection domain of FTD-mutant hTau is able to decrease protein synthesis and ribosomal complex formation faster and to a more pronounced level than non-mutant hTau.

While it is likely that other toxic effects of tau pathology contribute to decreased protein synthesis and ribosomal biogenesis, there is growing evidence to suggest that tau interferes with these processes directly, with tau being observed to be able to decrease protein synthesis and ribosomal complex formation even when isolated from the cell [19, 46]. Tau may also impair ribosomal biogenesis directly through its interactions with the nucleus, as ribosomal biogenesis is initiated at the nucleolus and requires ribosomal proteins to be imported into the nucleus [52]. Indeed, tau has been observed to relocate to the nucleolus [53] and to block nuclo-cytoplasmic transport in tauopathy models [54], which could impair ribosomal biogenesis.

## Conclusions

In our study, we have demonstrated that expression of either non-mutant or FTD-mutant human tau can impair the biogenesis of the 60S ribosomal subunit and that this effect is facilitated through the N-terminal projection domain of hTau. We also showed that FTD-mutant hTau expression decreases the abundance of specific ribosomal proteins, with these effects becoming more severe the longer tau was present. Together, our findings reveal that ribosomal function is impaired in tauopathy; however, whether ribosomal function is similarly impaired in other neurodegenerative diseases remains to be determined.

## Supplementary Information

**Additional File 1: Supplementary Fig. S1.** STRING network analysis reveals clusters of proteins involved in translation altered in K3 primary neurons. (A) Volcano plot of proteins quantified in K3 primary neurons. Label-free quantitative mass spectrometry was used to quantify the levels of 2,242 proteins from K3 and WT primary cortical neurons. The 160 proteins decreased in abundance (FC≤0.66, p-value ≤0.05) are coloured in blue, whereas the 22 proteins increased in abundance (FC≥1.5, p-value ≤0.05) are coloured in red. Ribosomal proteins which were significantly altered in abundance are labelled. (B) STRING network analysis of the 182 proteins significantly altered in K3 primary neurons. Interactions with a STRING score ≥ 0.7 are shown. Node size and colour are linearly related to fold-change. REACTOME analysis of the identified clusters revealed that they were associated with the processes of cap-dependent translation initiation, mRNA splicing, clathrin-mediated endocytosis, and Golgi-to-ER retrograde transport.

**Additional File 2: Supplementary Fig. S2.** Select ribosomal protein mRNAs show a trend of increase in abundance after 7 days of FTD-mutant hTau expression. (A) Cells expressing EGFP, hTau-EGFP, P301L-hTau-EGFP or K369I-hTau-EGFP for 24h revealed no significant change in the mRNA levels of Rpl5, Rps14, Rps6 or Rpl22, as quantified using qRT-PCR. n=3 wells. (B) Cells expressing EGFP, hTau-EGFP, P301L-hTau-EGFP or K369I-hTau-EGFP for 7 days and selected using neomycin. n=3-5 wells.

**Additional File 3: Supplementary Fig. S3.** Ribosomal biogenesis is also decreased by expression of K369I-hTau in the 2N4R isoform. 2N4R K369I-hTau reduces 60S ribosomal biogenesis and the abundance of monosomes and polysomes after 24 hours of expression. HEK293 cells transfected with Emerald, 2N4R hTau and 2N4R K369I-EGFP were treated with 100 µg/ml CHX for 5 minutes in order to prevent the dissociation of bound ribosomes from mRNA and following lysis, ribosomal complexes were separated on a 10–50% linear sucrose gradient via ultracentrifugation. 40S and 60S ribosomal subunit, along with monosome and polysomes were detected via their absorbance at 260 nm, with the area under the curve (AUC) of these peaks being used for quantification. Polysome, monosome and 60S abundance was only decreased by K369I-hTau expression, with non-mutant hTau expressing cells being unchanged compared to Emerald control. n= 3 wells, one-way ANOVA, Tukey’s MCT.

**Additional File 4:** List of ribosomal protein abundance in K3 primary neurons. List of ribosomal proteins quantified in primary cortical neurons cultured from K369I mice, with their fold-change relative to WT controls. Neurons from individual pups were cultured until DIV18 before label-free quantitative mass spectrometry was used to analyse the abundance of 80 ribosomal proteins. Data includes UniProt accession number, description of the RP, the number of unique peptides used in quantification, the fold-change compared to WT average for each animal, the fold-change in K3 pups vs WT control and the p-value result of an unpaired t-test comparison. Significantly altered proteins are coloured and shown with bold text.

**Additional File 5:** Label-free quantitative mass spectrometry analysis of K3 primary neurons. Quantification of proteins in primary cortical neurons cultured from K369I mice; fold-change relative to WT controls. Neurons from individual pups were cultured until DIV18 before label-free quantitative mass spectrometry was used to analyze the abundance of 2242 proteins. Data includes UniProt accession number, gene name, protein description, fold-change compared to WT average for each animal, fold-change in K3 pups and the p-value result of an unpaired t-test comparison. Significantly altered proteins are shown in colour.

## Data Availability

All data generated or analysed during this study are included in this published article and its additional files.

## Abbreviations

AD: Alzheimer’s disease
AHA: Azidohomoalanine
AU: Arbitrary units
AUC: Area under the curve
CHX: Cycloheximide
*C-term* hTau: Carboxy-terminal human tau construct
DIV: Days in vitro
DMEM: Dulbecco's modified Eagle's medium
DTT: 1,4-Dithiothreitol
EGFP: Enhanced green fluorescent protein
eIF: Eukaryotic initiation factor
FBS: Fetal bovine serum
FTD: Frontotemporal dementia
FUNCAT: Fluorescent non‐canonical amino acid tagging
HEK293 cells: Human embryonic kidney 293 cells
hTau: Human tau
IgG: Immunoglobulin G
MAPT: Microtubule-associated protein tau
MCT: Multiple comparison test
MRPL: Mitochondrial ribosomal protein large subunit
*MTBR* hTau: Microtubule binding region human tau construct
mTOR: Mammalian target of rapamycin
Nano-LC MS/MS: Nano‐liquid chromatography tandem mass spec
PBS: Phosphate‐buffered saline
PDL: Poly-D-lysine
*Proj-dom* hTau: N-terminal projection domain human tau construct
RIPA: Radioimmunoprecipitation assay
RP: Ribosomal protein
RPL: Ribosomal protein large subunit
RPS: Ribosomal protein small subunit
SFPQ: Splicing factor proline and glutamine rich
qRT-PCR: Semi-quantitative real-time polymerase chain reaction
TBS: Tris-buffered saline
TIA1: T cell intracellular antigen 1
WB: Western blott
WT: Wild-type

## References

[1] Frandemiche ML, De Seranno S, Rush T, Borel E, Elie A, Arnal I (2014). Activity-dependent tau protein translocation to excitatory synapse is disrupted by exposure to amyloid-beta oligomers. J Neurosci..

[2] Padmanabhan P, Martínez-Mármol R, Xia D, Götz J, Meunier FA (2019). Frontotemporal dementia mutant Tau promotes aberrant Fyn nanoclustering in hippocampal dendritic spines. Elife..

[3] Pallas-Bazarra N, Jurado-Arjona J, Navarrete M, Esteban JA, Hernández F, Ávila J (2016). Novel function of Tau in regulating the effects of external stimuli on adult hippocampal neurogenesis. EMBO J..

[4] Kneynsberg A, Combs B, Christensen K, Morfini G, Kanaan NM (2017). Axonal Degeneration in Tauopathies: Disease Relevance and Underlying Mechanisms. Front Neurosci..

[5] Götz J, Bodea LG, Goedert M (2018). Rodent models for Alzheimer disease. Nat Rev Neurosci..

[6] Bodea LG, Eckert A, Ittner LM, Piguet O, Götz J (2016). Tau physiology and pathomechanisms in frontotemporal lobar degeneration. J Neurochem..

[7] Morris M, Knudsen GM, Maeda S, Trinidad JC, Ioanoviciu A, Burlingame AL (2015). Tau post-translational modifications in wild-type and human amyloid precursor protein transgenic mice. Nat Neurosci..

[8] Götz J, Xia D, Leinenga G, Chew YL, Nicholas HR (2013). What renders tau toxic. Front Neurol..

[9] Ghetti B, Oblak AL, Boeve BF, Johnson KA, Dickerson BC, Goedert M (2015). Frontotemporal dementia caused by microtubule-associated protein tau gene ( MAPT ) mutations: a chameleon for neuropathology and neuroimaging. Neuropathol Appl Neurobiol..

[10] Naseri NN, Wang H, Guo J, Sharma M, Luo W (2019). The complexity of tau in Alzheimer’s disease. Neurosci Lett..

[11] Woollacott IOC, Rohrer JD (2016). The clinical spectrum of sporadic and familial forms of frontotemporal dementia. J Neurochem..

[12] Alberici A, Gobbo C, Panzacchi A, Nicosia F, Ghidoni R, Benussi L (2004). Frontotemporal dementia: Impact of P301L tau mutation on a healthy carrier. J Neurol Neurosurg Psychiatry..

[13] Neumann M, Schulz-Schaeffer W, Crowther RA, Smith MJ, Spillantini MG, Goedert M (2001). Pick’s disease associated with the novel tau gene mutation K369I. Ann Neurol..

[14] Benetatos J, Bennett RE, Evans HT, Ellis SA, Hyman BT, Bodea LG (2020). PTEN activation contributes to neuronal and synaptic engulfment by microglia in tauopathy. Acta Neuropathol..

[15] Blackmore T, Meftah S, Murray TK, Craig PJ, Blockeel A, Phillips K (2017). Tracking progressive pathological and functional decline in the rTg4510 mouse model of tauopathy. Alzheimers Res Ther..

[16] Wang Y, Mandelkow E (2016). Tau in physiology and pathology. Nat Rev Neurosci..

[17] Evans HT, Benetatos J, van Roijen M, Bodea LG, Götz J (2019). Decreased synthesis of ribosomal proteins in tauopathy revealed by non-canonical amino acid labelling. EMBO J..

[18] Koren SA, Hamm MJ, Meier SE, Weiss BE, Nation GK, Chishti EA (2019). Tau drives translational selectivity by interacting with ribosomal proteins. Acta Neuropathol..

[19] Meier S, Bell M, Lyons DN, Rodriguez-Rivera J, Ingram A, Fontaine SN (2016). Pathological tau promotes neuronal damage by impairing ribosomal function and decreasing protein synthesis. J Neurosci..

[20] Li C, Götz J (2017). Somatodendritic accumulation of Tau in Alzheimer’s disease is promoted by Fyn-mediated local protein translation. EMBO J..

[21] Yoon BC, Zivraj KH, Holt CE (2009). Local translation and mRNA trafficking in axon pathfinding. Results Probl Cell Differ..

[22] Zukin RS, Richter JD, Bagni C (2009). Signals, synapses, and synthesis: how new proteins control plasticity. Front Neural Circuits..

[23] Piochon C, Kano M, Hansel C (2016). LTD-like molecular pathways in developmental synaptic pruning. Nat Neurosci..

[24] Evans HT, Bodea LG, Götz J (2020). Cell-specific non-canonical amino acid labelling identifies changes in the de novo proteome during memory formation. Elife..

[25] Davis HP, Squire LR (1984). Protein synthesis and memory: A review. Psychol Bull..

[26] Lopez J, Gamache K, Schneider R, Nader K (2015). Memory retrieval requires ongoing protein synthesis and NMDA receptor activity-mediated AMPA receptor trafficking. J Neurosci..

[27] Apicco DJ, Ash PEA, Maziuk B, LeBlang C, Medalla M, Al Abdullatif A (2018). Reducing the RNA binding protein TIA1 protects against tau-mediated neurodegeneration in vivo. Nat Neurosci..

[28] Ke Y, Dramiga J, Schütz U, Kril JJ, Ittner LM, Schröder H (2012). Tau-mediated nuclear depletion and cytoplasmic accumulation of SFPQ in Alzheimer’s and Pick’s disease. PLoS One..

[29] Ittner LM, Fath T, Ke YD, Bi M, van Eersel J, Li KM (2008). Parkinsonism and impaired axonal transport in a mouse model of frontotemporal dementia. Proc Natl Acad Sci..

[30] Bodea LG, Evans HT, Van der Jeugd A, Ittner LM, Delerue F, Kril J (2017). Accelerated aging exacerbates a pre-existing pathology in a tau transgenic mouse model. Aging Cell..

[31] Bodea LG, Wang Y, Linnartz-Gerlach B, Kopatz J, Sinkkonen L, Musgrove R (2014). Neurodegeneration by Activation of the Microglial Complement-Phagosome Pathway. J Neurosci..

[32] Pringle ES, McCormick C, Cheng Z (2019). Polysome Profiling Analysis of mRNA and Associated Proteins Engaged in Translation. Curr Protoc Mol Biol..

[33] Bader GD, Hogue CWV (2003). An automated method for finding molecular complexes in large protein interaction networks. BMC Bioinformatics..

[34] Ittner LM, Ke YD, Götz J (2009). Phosphorylated tau interacts with c-Jun N-terminal Kinase-interacting Protein 1 (JIP1) in Alzheimer disease. J Biol Chem..

[35] Evans HT, Blackmore D, Götz J, Bodea LG (2021). De novo proteomic methods for examining the molecular mechanisms underpinning long-term memory. Brain Res Bull..

[36] Pandit R, Leinenga G, Götz J (2019). Repeated ultrasound treatment of tau transgenic mice clears neuronal tau by autophagy and improves behavioral functions. Theranostics..

[37] Dörrbaum AR, Kochen L, Langer JD, Schuman EM (2018). Local and global influences on protein turnover in neurons and glia. Elife..

[38] Doudna JA, Rath VL (2002). Structure and function of the Eukaryotic Ribosome. Cell..

[39] Garcia-Esparcia P, Hernández-Ortega K, Koneti A, Gil L, Delgado-Morales R, Castaño E (2015). Altered machinery of protein synthesis is region- and stage-dependent and is associated with α-synuclein oligomers in Parkinson’s disease. Acta Neuropathol Commun..

[40] Hernández-Ortega K, Garcia-Esparcia P, Gil L, Lucas JJ, Ferrer I (2016). Altered machinery of Protein synthesis in Alzheimer’s: from the Nucleolus to the Ribosome. Brain Pathol..

[41] Bernabò P, Tebaldi T, Groen EJN, Lane FM, Perenthaler E, Mattedi F (2017). In vivo translatome profiling in spinal muscular atrophy reveals a role for SMN Protein in Ribosome biology. Cell Rep..

[42] Nandagopal N, Roux PP (2015). Regulation of global and specific mRNA translation by the mTOR signaling pathway. Translation..

[43] Iadevaia V, Liu R, Proud CG (2014). mTORC1 signaling controls multiple steps in ribosome biogenesis. Semin Cell Dev Biol..

[44] Mayer C, Grummt I (2006). Ribosome biogenesis and cell growth: mTOR coordinates transcription by all three classes of nuclear RNA polymerases. Oncogene..

[45] Tramutola A, Triplett JC, Di Domenico F, Niedowicz DM, Murphy MP, Coccia R (2015). Alteration of mTOR signaling occurs early in the progression of Alzheimer disease (AD): analysis of brain from subjects with pre-clinical AD, amnestic mild cognitive impairment and late-stage AD. J Neurochem..

[46] Banerjee S, Ferdosh S, Ghosh AN, Barat C (2020). Tau protein- induced sequestration of the eukaryotic ribosome: Implications in neurodegenerative disease. Sci Rep..

[47] Zabik NL, Imhof MM, Martic-Milne S (2017). Structural evaluations of tau protein conformation: methodologies and approaches. Biochem Cell Biol..

[48] Di Primio C, Quercioli V, Siano G, Rovere M, Kovacech B, Novak M (2017). The Distance between N and C Termini of Tau and of FTDP-17 Mutants Is Modulated by Microtubule Interactions in Living Cells. Front Mol Neurosci..

[49] Jeganathan S, Hascher A, Chinnathambi S, Biernat J, Mandelkow EM, Mandelkow E (2008). Proline-directed pseudo-phosphorylation at AT8 and PHF1 epitopes induces a compaction of the paperclip folding of tau and generates a pathological (MC-1) conformation. J Biol Chem..

[50] Kawasaki R, Tate S (2020). Impact of the hereditary P301L mutation on the correlated conformational dynamics of human tau protein revealed by the paramagnetic relaxation enhancement NMR experiments. Int J Mol Sci..

[51] Barbier P, Zejneli O, Martinho M, Lasorsa A, Belle V, Smet-Nocca C (2019). Role of tau as a microtubule-associated protein: structural and functional aspects. Front Aging Neurosci..

[52] Baßler J, Hurt E (2019). Eukaryotic ribosome assembly. Annu Rev Biochem..

[53] Maina MB, Bailey LJ, Doherty AJ, Serpell LC (2018). The involvement of Aβ42 and tau in nucleolar and protein synthesis machinery dysfunction. Front Cell Neurosci..

[54] Eftekharzadeh B, Daigle JG, Kapinos LE, Coyne A, Schiantarelli J, Carlomagno Y (2018). Tau Protein Disrupts Nucleocytoplasmic Transport in Alzheimer’s Disease. Neuron.

