# Supplementary material for: Altered ribosomal function and protein synthesis caused by tau
Source: Acta Neuropathol Commun. 2021 Jun 19;9:110. doi: 10.1186/s40478-021-01208-4 (PMC8214309; doi:10.1186/s40478-021-01208-4)

Supplementary Figure 1

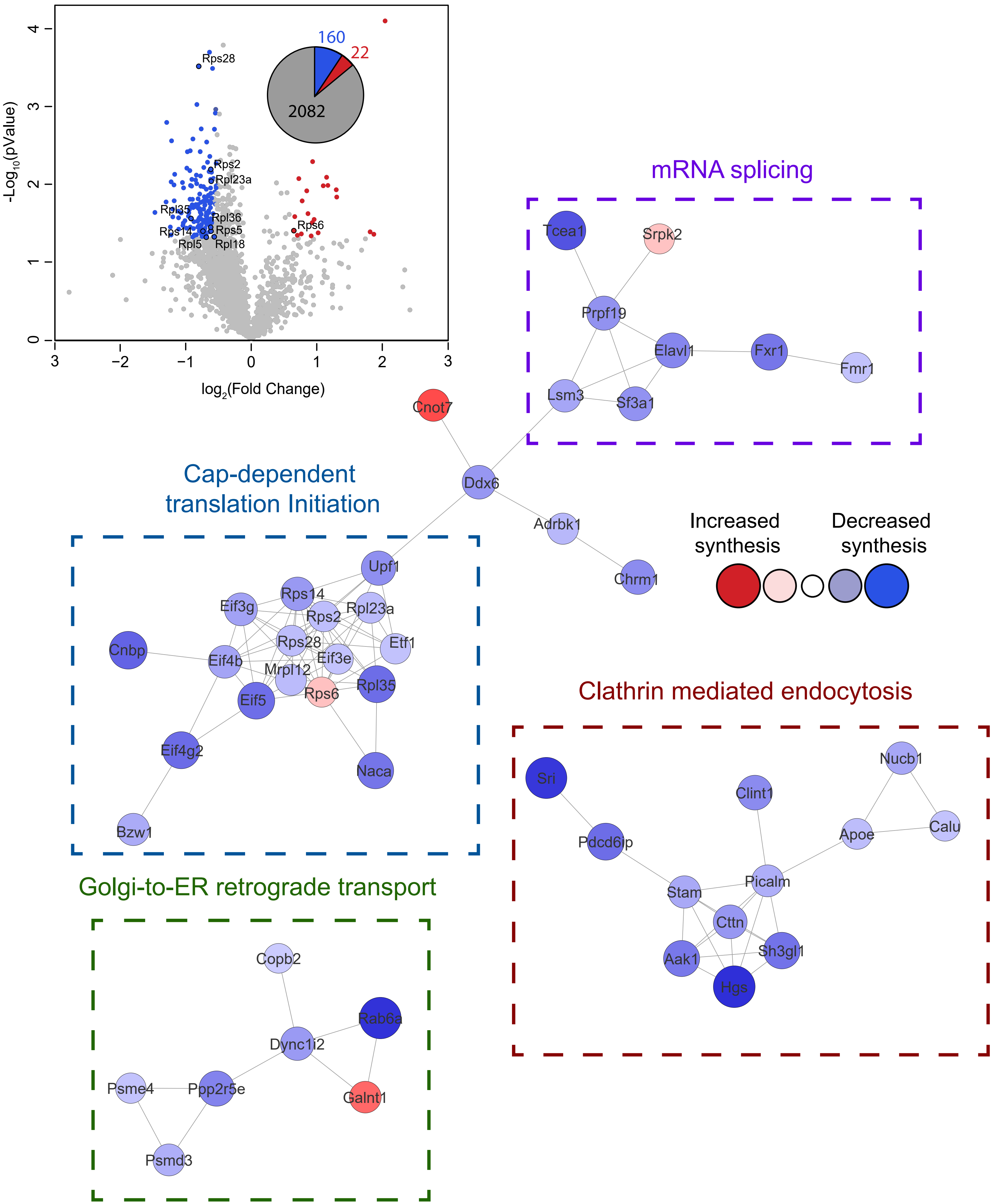

Supplement: Supplementary file 1 — Additional File 1: Supplementary Fig. S1. STRING network analysis reveals clusters of proteins involved in translation altered in K3 primary neurons. (A) Volcano plot of proteins quantified in K3 primary neurons. Label-free quantitative mass spectrometry was used to quantify the levels of 2,242 proteins from K3 and WT primary cortical neurons. The 160 proteins decreased in abundance (FC≤0.66, p-value ≤0.05) are coloured in blue, whereas the 22 proteins increased in abundance (FC≥1.5, p-value ≤0.05) are coloured in red. Ribosomal proteins which were significantly altered in abundance are labelled. (B) STRING network analysis of the 182 proteins significantly altered in K3 primary neurons. Interactions with a STRING score ≥ 0.7 are shown. Node size and colour are linearly related to fold-change. REACTOME analysis of the identified clusters revealed that they were associated with the processes of cap-dependent translation initiation, mRNA splicing, clathrin-mediated endocytosis, and Golgi-to-ER retrograde transport. [file 40478_2021_1208_MOESM1_ESM.pdf]
