# Supplementary figures and images for: Altered ribosomal function and protein synthesis caused by tau
Source: Acta Neuropathol Commun. 2021 Jun 19;9:110. doi: 10.1186/s40478-021-01208-4 (PMC8214309; doi:10.1186/s40478-021-01208-4)

## Supplementary Figure 2

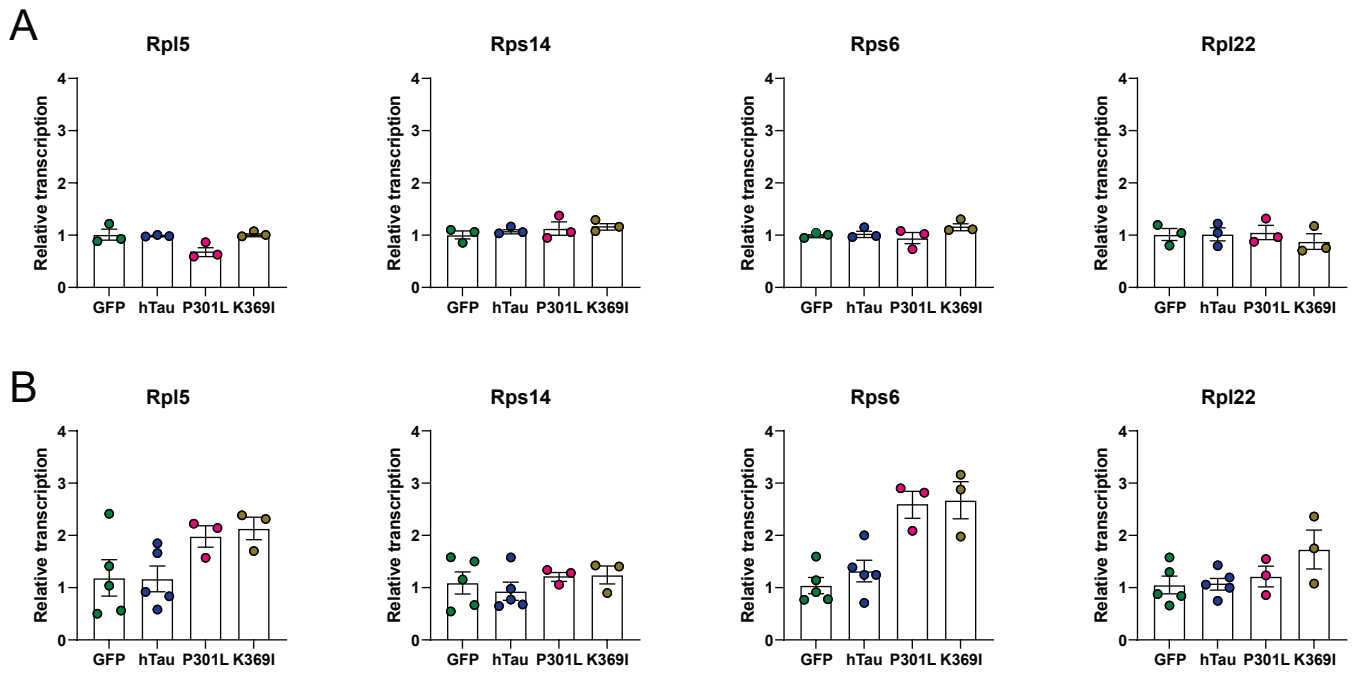

Supplement: Supplementary file 2 — Additional File 2: Supplementary Fig. S2. Select ribosomal protein mRNAs show a trend of increase in abundance after 7 days of FTD-mutant hTau expression. (A) Cells expressing EGFP, hTau-EGFP, P301L-hTau-EGFP or K369I-hTau-EGFP for 24h revealed no significant change in the mRNA levels of Rpl5, Rps14, Rps6 or Rpl22, as quantified using qRT-PCR. n=3 wells. (B) Cells expressing EGFP, hTau-EGFP, P301L-hTau-EGFP or K369I-hTau-EGFP for 7 days and selected using neomycin. n=3-5 wells. [file 40478_2021_1208_MOESM2_ESM.pdf]

**Supplementary Figure 3**

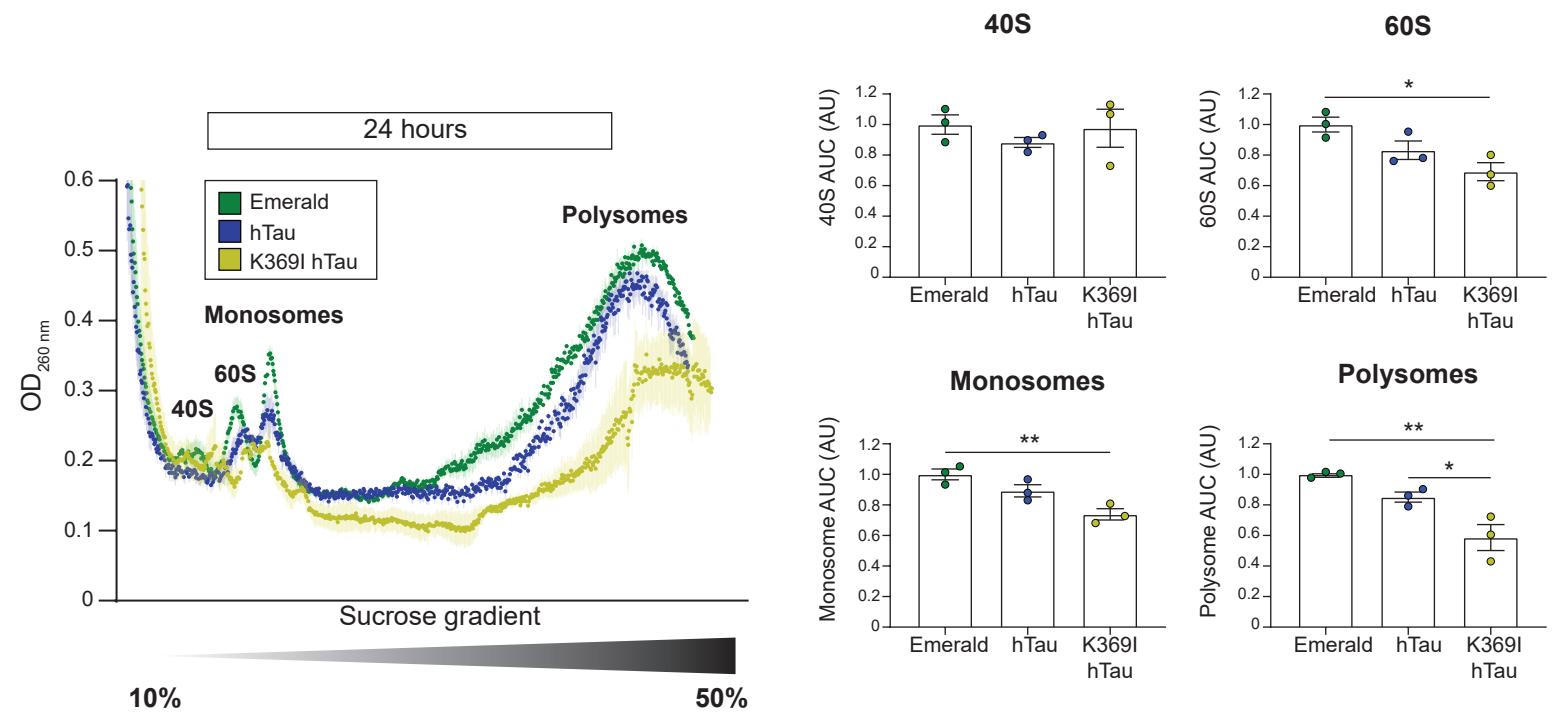

Supplement: Supplementary file 3 — Additional File 3: Supplementary Fig. S3. Ribosomal biogenesis is also decreased by expression of K369I-hTau in the 2N4R isoform. 2N4R K369I-hTau reduces 60S ribosomal biogenesis and the abundance of monosomes and polysomes after 24 hours of expression. HEK293 cells transfected with Emerald, 2N4R hTau and 2N4R K369I-EGFP were treated with 100 µg/ml CHX for 5 minutes in order to prevent the dissociation of bound ribosomes from mRNA and following lysis, ribosomal complexes were separated on a 10–50% linear sucrose gradient via ultracentrifugation. 40S and 60S ribosomal subunit, along with monosome and polysomes were detected via their absorbance at 260 nm, with the area under the curve (AUC) of these peaks being used for quantification. Polysome, monosome and 60S abundance was only decreased by K369I-hTau expression, with non-mutant hTau expressing cells being unchanged compared to Emerald control. n= 3 wells, one-way ANOVA, Tukey’s MCT. [file 40478_2021_1208_MOESM3_ESM.pdf]
